# Supplementary material for: Rho1 activation recapitulates early gastrulation events in the ventral, but not dorsal, epithelium of Drosophila embryos
Source: eLife. 2020 Nov 17;9:e56893. doi: 10.7554/eLife.56893 (PMC7717907; doi:10.7554/eLife.56893)
Supplement: Supplementary file 1. — References cited in this file: Guntas et al., 2015; Munjal et al., 2015; Valbuena et al., 2020; Wagner and Glotzer, 2016; Wenzl et al., 2010. [file elife-56893-supp1.pdf]

| Plasmid Generated | Fragment Source | Backbone Source |
|---|---|---|
| pMT>Stargazin-GFP*-LOVSsrA (* represents inactivation of fluorphore with Y66C mutation.) | Stargazin-GFP amplified from Stargazin-GFP-LOVpep (***Wagner and Glotzer, 2016***) LOVSsrA amplified from Venus-iLID-CAAX (***Guntas et al., 2015***) (Addgene: 60411) GFP silenced by site-directed mutagenesis (Y66C) | pMT>Gal4 (***Klueg et al., 2002***) |
| pMT>Stargazin-GFP*-LOV(I427V)SsrA | N/A | pMT>Stargazin-GFP*-LOVSsrA w/ site directed mutagenesis |
| pUbi>Stargazin-GFP*-LOVSsrA | Stargazin-GFP*-LOVSsrA amplified from pMT>Stargazin-GFP*-LOVSsrA | pUbi-stop-mCD8GFP (Contains attB site) |
| pUbi>Stargazin-GFP*-LOV(I427V)SsrA | Stargazin-GFP*-LOV(I427V)SsrA amplified from pMT>Stargazin-GFP*-LOV(I427V)SsrA | pUbi-stop-mCD8GFP (Contains attB site) |
| pUbi>SspB-GFP-LARG(DH) | LARG(DH) amplified from PDZx2-mCherry-LARG(DH) (***Wagner and Glotzer, 2016***) SspB amplified from tgRFPt-SspB(WT) (***Guntas et al., 2015***) (Addgene: 60415) | pUbi-stop-mCD8GFP (Contains attB site) |
| pMT>tagRFP-SspB | SspB amplified from tgRFPt-SspB(WT) (***Guntas et al., 2015***) (Addgene: 60415) | pMT>Gal4 |
| pUbi>SspB-mScar | SspB amplified from pMT>tagRFP-SspB mScar amplified from pmScarlet-C1 (Addgene: 85042) | pUbi-stop-mCD8GFP (Contains attB site) |
| pUbi>SspB-GFP-RhoGEF2(DHPH) | RhoGEF2(DHPH) amplified from genomic prep of Sp/CyO; UASp>RFP-RhoGEF2/TM3 (***Wenzl et al., 2010***) | pUbi-SspB-GFP-LARG(DH) (Replace LARG(DH)) (Contains attB site) |
| pUbi>SspB-GFP-RhoGEF2(DHPH-F1044A,I1046E) | N/A | pUbi>SspB-GFP-RhoGEF2(DHPH) w/ site directed mutagenesis |
| pUbi>mCherry-Anillin(RBD) | Anillin(RBD) amplified from pUbi>mEGFP-Anillin(RBD) (***Munjal et al., 2015***) mCherry amplified from pm-Cherry2B (***Valbuena et al., 2020***) | pUbi-stop-mCD8GFP (Contains attB site) |
