# Supplementary material for: Rho1 activation recapitulates early gastrulation events in the ventral, but not dorsal, epithelium of Drosophila embryos
Source: eLife. 2020 Nov 17;9:e56893. doi: 10.7554/eLife.56893 (PMC7717907; doi:10.7554/eLife.56893)
Supplement: Supplementary file 2. [file elife-56893-supp2.pdf]

Figure	Genotype	Replicates
Figure 1b	*SspB-mScarlet ; Stargazin-GFP*-LOVSsrA*	4/4 embryos
Figure 1d	*SspB-GFP-LARG(DH) ; Stargazin-GFP*-LOVSsrA, Sqh-mCherry / Stargazin-GFP*-LOVSsrA*	5/5 embryos
Figure 2	*SspB-GFP-LARG(DH); Stargazin-GFP*-LOVSsrA, Gap43-mCherry*	3/3 embryos; 463 cells (b-d);<br>203 cells (e)
Figure 3	*SspB-GFP-LARG(DH) ; Stargazin-GFP*-LOVSsrA, Sqh-mCherry / Stargazin-GFP*-LOVSsrA*	7/7 embryos (a)<br>5/5 embryos (b)
Figure 4	*SspB-GFP-LARG(DH); Stargazin-GFP*-LOVSsrA, Gap43-mCherry*	2/2 embryos; 7/8 cells (a)<br>4/4 embryos; 444 cells (b)<br>4/4 embryos; 288 cells (c)
Figure 5	Δ *halo AJ twist*^{EY53R12} *; SspB-GFP-LARG(DH), Stargazin-GFP*-LOVSsrA, Gap43-mCherry*<br>*dl*^{1} *cn*^{1} *sca*^{1} *; SspB-GFP-LARG(DH), Stargazin-GFP*-LOVSsrA, Gap43-mCherry*<br>F1 of: *P(mat-tub-Gal4)mat67 / SspB-GFP-LARG(DH); SspB-GFP-LARG(DH),*<br>*Stargazin-GFP*-LOVSsrA, Gap43-mCherry / Stargazin-GFP*-LOVSsrA UAS>RhoGEF2 shRNA*<br>*SspB-GFP-LARG(DH); Stargazin-GFP*-LOVSsrA, Gap43-mCherry (square zone)*	3/3 embryos ; 189 cells<br>4/4 embryos ; 343 cells<br>5/5 embryos ; 375 cells<br>(ventrally oriented)<br>5/5 embryos; 436 cell<br>(dorsally oriented)<br>5/5 embryos ; 239 cells
Figure 6	*SspB-GFP-LARG(DH) ; Stargazin-GFP*-LOVSsrA, Sqh-Ch*	3/5 embryos
Figure 7	*SspB-GFP-LARG(DH); Stargazin-GFP*-LOVSsrA, Gap43-mCherry*	4/4 embryos (a)<br>4/4 embryos (b)<br>407 cells, dorsal (c)<br>298 cells, ventral (c)
