# Supplementary material for: Rho1 activation recapitulates early gastrulation events in the ventral, but not dorsal, epithelium of Drosophila embryos
Source: eLife. 2020 Nov 17;9:e56893. doi: 10.7554/eLife.56893 (PMC7717907; doi:10.7554/eLife.56893)
Supplement: Supplementary file 3. [file elife-56893-supp3.pdf]

Figure	Genotype	Replicates
Figure 1–Figure Supplement 1a	*SspB-GFP-LARG(DH); Stargazin-GFP*-LOVSsrA, Sqh-mCherry*	2/2 pupae
Figure 1–Figure Supplement 1b		4/4 egg chambers
Figure 1–Figure Supplement 1c		5/5 (left), 3/3 (right) wing discs
Figure 1–Figure Supplement 1d		2 CNSs ; 6 Neuroblasts
Figure 1–Figure Supplement 2	*SspB-GFP-LARG(DH); Stargazin-GFP*-LOVSsrA, Sqh-mCherry*	3/3 wing discs
Figure 1–Figure Supplement 3a-top	Transfection: tagRFP-SspB + Stargazin-GFP*-LOVSsrA	3/3 cells
Figure 1–Figure Supplement 3a-bottom	Transfection: tagRFP-SspB + Stargazin-GFP*-LOV(I427V)SsrA	3/3 cells
Figure 1–Figure Supplement 3c-top	*SspB-GFP-LARG(DH) Ubi>mCherry-Anillin(RB) ; Stargazin-GFP*-LOVSsrA*	3 brains ; 16 neuroblasts
Figure 1–Figure Supplement 3c-bottom	*SspB-GFP-LARG(DH) Ubi>mCherry-Anillin(RB); Stargazin-GFP*-LOV(I427V)SsrA*	4 brains ; 19 neuroblasts
Figure 2–Figure Supplement 1	see ***Figure 2***b-d & ***Figure 4***b-c	see ***Figure 2***b-d & ***Figure 4***b-c
Figure 2–Figure Supplement 2	F1 of: *Sqh-GFP/Cyo ; Gap43-Ch/TM6*	3/3 embryos; 436 cells
Figure 3–Figure Supplement 1	*SspB-GFP-LARG(DH) ; Stargazin-GFP*-LOVSsrA, Sqh-Ch / Stargazin-GFP*-LOVSsrA*	3/3 embryos
Figure 3–Figure Supplement 2	*SspB-GFP-LARG(DH) ; Stargazin-GFP*-LOVSsrA, Sqh-Ch / Stargazin-GFP*-LOVSsrA*	4/5 embryos (dorsal)
Figure 3–Figure Supplement 3	F1 of: *P(mat-tub-Gal4)mat67 / SspB-GFP-LARG(DH); SspB-GFP-LARG(DH),*<br>*Stargazin-GFP*-LOVSsrA, Sqh-Ch / Stargazin-GFP*-LOVSsrA UAS>RhoGEF2 shRNA*	4/4 embryos (dorsal)<br>5/5 embryos (ventral)
Figure 4–Figure Supplement 1	see ***Figure 4*** & ***Figure 5***	see ***Figure 4*** & ***Figure 5***
Figure 4–Figure Supplement 2	see ***Figure 4*** & ***Figure 5***	see ***Figure 4*** & ***Figure 5***
Figure 4–Figure Supplement 3	*SspB-GFP-LARG(DH) ; Stargazin-GFP*-LOVSsrA, Gap43-Ch*	4/4 embryos
Figure 5–Figure Supplement 1	See ***Figure 5***	See ***Figure 5***
