# Supplementary material for: Rho1 activation recapitulates early gastrulation events in the ventral, but not dorsal, epithelium of Drosophila embryos
Source: eLife. 2020 Nov 17;9:e56893. doi: 10.7554/eLife.56893 (PMC7717907; doi:10.7554/eLife.56893)
Supplement: Supplementary file 4. [file elife-56893-supp4.pdf]

Figure	Microscope, Wavelength	Activation Protocol	Total Activation Time
Figure 1b	LSM880, 488nm	Every 20 sec	1 min 40 sec
Figure 1d	Spinning Disc, 488nm	Global (1000ms) every 20 sec	1 min
Figure 3a-b	LSM880, 488nm	Every 20 sec	4min
Figure 4a-b	LSM880, 488nm	Every 20 sec	4 min
Figure 5	LSM880, 488nm	Every 20 sec	4 min
Figure 6	LSM880, 488nm	Every 20 sec	1 min 20 sec
Figure 7a-b	LSM880, 488nm	Every 20 sec	4 min
