# Supplementary material for: Rho1 activation recapitulates early gastrulation events in the ventral, but not dorsal, epithelium of Drosophila embryos
Source: eLife. 2020 Nov 17;9:e56893. doi: 10.7554/eLife.56893 (PMC7717907; doi:10.7554/eLife.56893)
Supplement: Supplementary file 5. [file elife-56893-supp5.pdf]

| Figure | Microscope, Wavelength | Activation Protocol | Total Activation Time |
|---|---|---|---|
| ***Figure 1–Figure Supplement 1***a | Spinning Disc, 488nm | Global every 15 sec | 30 sec |
| ***Figure 1–Figure Supplement 1***b | Spinning Disc, 488nm | Global every 5 sec | 15 sec |
| ***Figure 1–Figure Supplement 1***c | LSM880, 488nm | Every 20 sec | 2:01 min (left)<br>1:40 (right) |
| ***Figure 1–Figure Supplement 1***d | LSM880, 488nm | Every 20 sec | 1 min |
| ***Figure 1–Figure Supplement 2*** | LSM880, 405nm | Every 20 sec<br>(See indicated percent transmittance) | 2 min 20 sec |
| ***Figure 1–Figure Supplement 3***a-b | Spinning Disc, 488nm | Global every 5 sec | 5 sec |
| ***Figure 1–Figure Supplement 3***c | Spinning Disc, 488nm | Global every 5 sec | 20 sec |
| ***Figure 3–Figure Supplement 1*** | LSM880, 488nm | Every 20 sec | 4 min 40s |
| ***Figure 3–Figure Supplement 2*** | LSM880, 488nm | Every 20 sec | 6 min 35 sec (left) |
| ***Figure 3–Figure Supplement 3*** | LSM880, 488nm | Every 20 sec | 1 min 40 sec |
| ***Figure 4–Figure Supplement 1*** | See ***Figure 4*** & ***Figure 5*** | See ***Figure 4*** & ***Figure 5*** | See ***Figure 4*** & ***Figure 5*** |
| ***Figure 4–Figure Supplement 2*** | See ***Figure 4*** & ***Figure 5*** | See ***Figure 4*** & ***Figure 5*** | See ***Figure 4*** & ***Figure 5*** |
| ***Figure 4–Figure Supplement 3*** | LSM880, 488nm | Every 20 sec | 1 min 40 sec |
| ***Figure 5–Figure Supplement 1*** | See ***Figure 5*** | See ***Figure 5*** | See ***Figure 5*** |
